# On the Progress of Anatomy and Surgery during the Present Century

**Published:** 1864-08

**Authors:** Wm. Fergusson


deeds inspire veneration, held sway in the great metropolis. In Glasgow, John Burns labored, I may say alone, in a field which had been previously occupied by himself and his worthy brother Allan. In Edinburgh, the reputation of the Monros gave high character to the anatomical and pathological aspect of surgery, and the family reputation was maintained by the third of the name. The brilliancy of John Bell had in the early part of the century given great *éclat* to the school (which was enhanced by his brother Charles, whose name may be honorably included amongst the worthies of London, at the time I speak of,) and the solid worth of Benjamin Bell had given a high character to Edinburgh surgery.

About this date the field of surgical practice in the northern metropolis was held by gentlemen of high social and professional stamp, but they were neither professed teachers nor long-experienced hospital surgeons. Each had served a few years only as full surgeon in the Royal Infirmary. One (Mr. Wishart) had published translations of Scarpa's works on Aneurism and on Hernia, but others were unknown to more than local fame. From this list I may bring out and except the name of Russell, the author of an original and still standard work on Necrosis, and at that time revered as a surviving pupil of John Hunter. He was, moreover, the first professor of clinical surgery, and the only one bearing such a title in the United Kingdom. His position as a model surgeon was, however, by no means prominent, and the "pure" surgery of Edinburgh (as the term goes) was little different from that which might be found in any of the large provincial towns in Britain. There was no chair of surgery in the University. That of the College of Surgeons (which was shortly after abrogated) was held by a clever man, whose health and temperament prevented him taking a foremost rank in practical surgery, and there seemed little hope for a continuance of the reputation of this school, when suddenly there appeared on the scene three men whose labors have added substantially to the renown of the Scotch school, and whose names will be imperishably associated with the history of British surgery. These men were, John Lizars, Robert Liston, and James Syme. I trust that I may be pardoned for making pointed allusion to these surgeons but as it was from them that I gathered many of my own early views in surgery, I should not wish this opportunity to pass without giving them that honorable mention which, in my opinion, they richly deserve.

Mr. Syme still lives in active manhood, with a world-wide reputation second to none amongst living surgeons. It is considered unbecoming to say that of one yet active on the scene which may be said in after years. Modern surgery owes him much, as I shall show in future lectures. Eulogy might seem to partake of flattery, and for my present purpose it may be sufficient to state, that at the date referred to, this gentleman evinced all that energy of character and aptitude for clinical teaching and for practice for which he has since become so distinguished.

Mr. Liston's fame, at this date, particularly as an operator, was well righ as great as at any period of his comparatively short but brilliant career. In after years his soundness as a pathologist became more conspicuous; and the numerous valuable preparations in the museum of this College, which formed part of his collection, bear ample testimony to the greatness of his doings in practical surgery. Both he and Mr. Syme had already published those remarkable essays on Practical Amputation which, with the example set by their practice, went far to give that development to the flap operation since attained. Many circumstances contributed to give Mr. Liston early fame in Scotland. A well-developed frame, a broad forehead, a strongly marked, handsome countenance, indicative of great courage and decision, and an eye of piercing brilliancy and great expression, at once impressed those who sought his aid with a conviction of his powers. With these were associated a hand alike marvellous for its great size, its silent expressiveness, its vigorous firmness, its lightness, and its dexterity. It was aptly said of it by a distinguished lay contemporary, the late Lord Robertson—" If hard as iron and true as steel in the theatre of operation, it is soft as thistledown when applied to the throbbing pulse or aching brow." The remembrance of that hand is still fresh on my memory.

Some early operations of great magnitude and comparative novelty, aided by a certain amount of jealous opposition which merit is sure to call forth, brought Mr. Liston's fame impressively before the public; and among his achievements may be mentioned the successful removal of a scrotal tumor of more than forty pounds weight—the first operation of the kind ever performed in this country—and successful ligature of the subclavian, which had been essayed in vain by Ramsden and others in Britain.

When personal recollections have passed away, there will remain much to associate Mr. Liston's name with surgery, but the greatest features of his teaching powers will be forgotten.

With less than average facility of speech, he had a manner in all that he did before his pupils that produced the deepest impression; and there was a style in his operations which has had more influence in this department among a large number of pupils than has been produced, in as far as I can make out, by any other man in the history of surgery. Only those who have seen him can thoroughly appreciate what I now say.

Of Mr. Lizars there is now probably less known than of the two gentlemen just referred to; but his fame was great at the time. His folio work on anatomy, with which he incorporated most of his views on operative surgery, had contributed largely to his reputation. Initiated to the profession by John Bell, to whom he served a pupilage, he seemed to have imbibed some of the characteristics of that great surgeon. He was a very successful teacher, both of anatomy and surgery, an excellent pathologist, a brilliant and daring operator. His name will ever remain associated with the early history of modern operations on the upper jaw. He was the only man in Scotland who had placed a ligature on the innominata. The operation was unsuccessful, but it went far to prove, what was then not so well recognized as now, that secondary hemorrhage in such cases is more likely to come from the distal than from the proximal end of a tied vessel. He was the second to perform ovariotomy, and its practical originator in Britain. Like many pioneers in art and science, he was for this assailed by a certain amount of ridicule associated with vigorous opposition, and thus was thrown into abeyance an operation which, thirty years later, has produced as much excitement as has been associated with the early history of any great surgical proceeding. Whatever may be the fate of ovariotomy, the name of John Lizars must always remain associated with it.

I may be wrong, but the impression is strong on my mind, that an impulse to the more acccurate study of surgical anatomy arose coeval with the development of the Hunterian operation. Before I knew the profession, all the great arteries had been tied, from the superficial femoral to the abdominal aorta and innominata, on the principles of our great surgical philosopher. The surgical anatomy of the arteries had occupied the attention of many first-rate anatomists of the early part of this century; and whilst the operations in question were excitingly attractive, others were not overlooked, and hence surgical and regional anatomy took a wider field, and the works of Charles Bell, Abraham Colles, Astley Cooper, John Shaw, Hargrave, and of others, testified to the zeal and accuracy of surgeons in those times in anatomical pursuits having direct relation to their calling. It is an anecdote worth bearing in mind that when Astley Cooper was engaged in his great and interesting labors on hernia, nothing would satisfy him but a sight of the fact that the obturator artery might encircle the inner side of the neck of a crural hernia. The first preparation that gave this proof was in the museum of the famous teacher of anatomy in Edinburgh, John Barclay (now incorporated with the collection of the Royal College of Surgeons of that city), who actually forwarded it to London to satisfy the hesitation of the great surgeon. It was returned with most complimentary thanks; and this anatomical fact, now familiar to the simplest novice, was soon after made extensively known to the professional world.

There were manuals of anatomy in those days, written by men who have since held the highest professional positions, which really left little for the practical surgeon to desire; in fact, the subject was in a manner exhausted. Whatever was essayed as novel, seemed in reality but a repetition of something already done and known; and, with an occasional exception, there was little left for the modern anatomist but transcendentalism and minute observation. Investigations on ill-defined and obscurely developed quantities have, I fear, taken largely the place of wholesome surgical anatomy; and whilst I shall not go so far as to say that they are not of great value to the education of the practical surgeon, I may state that I have often felt inclined to protest against a system which seems to draw little or no distinction between this kind of so-called philosophy and that common-place, but common-sense, anatomy which is of essential service to the practical surgeon. With some it almost appears as if the bulk of the two thousandth part of an inch were of equal importance to the surgeon as the outlines of the sterno-mastoid or deltoid muscles; and with many it seems to be that there is really little or no difference of essential value between "blastema" and bone, "molecule" and muscle, "cytoblast" and cellular membrane!—nay, actually that once familiar term is now in some degree tabooed, and a man's acquirements are suspected if he does not use instead the modern one of "areolar tissue."

In surgical pathology, it was known that a person might live with an obliterated aorta, and might survive the loss of an upper or lower extremity. Inflammation with denudation of bone was commonly believed to necessitate amputation; and diseased joints with ulceration of cartilages, particularly if denoted by crepitation, were generally deemed incurable, excepting by removal of the limb. Tumors of enormous size were frequently met with, and the disease then familiarly known as fungus hæmatodes was more common than in the present day: in both instances doubtless from timidity on the part of those who feared to meddle with what the modern surgeon arrests in early progress. But a vast amount of important material had been accumulated by the practical men of the day, and the works of Lawrence on Hernia, Brodie on the Joints, Thomson on Inflammation, Hodgson on the Arteries, and Cooper on Dislocations, may be referred to as types of the most valuable and precise surgical pathology which had been given to the profession. Pupils and practitioners had for study and reference in surgery, and to some extent in anatomy, the standard works of Boyer, of Benjamin Bell, of John and Charles Bell, of Abernethy, and of Samuel Cooper, whose "First Lines" was for a long time the favorite text-book, and whose famous Dictionary has, perhaps, not been excelled even to the present day.

Some naval and military surgeons had contributed largely to our general knowledge. Besides the labors of Hunter and of John Bell in these departments, it is in accordance with the intended spirit of this lecture that I should refer to those of Veitch and Copland Hutchinson, of Larrey, of Hennen, and of Guthrie. Although I am myself disposed to take exception to some of the doctrines of these gentlemen as being invariably applicable to the practice of surgery in civil life, I willingly acknowledge the great merits of those who gave us so much information after the cessation of our wars with the first Napoleon, and that much additional material, of unquestionable novelty and value, has been added to our stores by the publication of the so-called military surgery of that eventful period.

In Smiles' "Lives of Engineers" a dozen or more of those who first worked in this noble science are told off, each with a brief, yet interesting memoir, comprised within a few pages; but as engineering has advanced in the progress of time, the works of Vermuyden and Myddleton, of Metcalf and Brindley, seem to be surpassed by those of Smeaton, Telford, and Rennie, until at last a whole volume is required for the life of the elder Stephenson. Were we to compute the progress of surgery in a similar manner, to what limit might the lives of the great surgeons not go? To look within the present century, volumes might be written, in which most of the names already mentioned would stand pre-eminent, and it would not be difficult to mark out many of the living generation with whom the progress of surgery is closely associated. It is the boast of those who live in the nineteenth century, that progress in all that pertains to civilization has been greater than in any similar period in history. I can not venture to claim for surgery the world-wide impression that has been made by steam, by electricity, by engineering, or by mechanics. Yet our art and science have not stood still. If there have been any changes and reforms in our laws and civil institutions for the improvement of our social atmosphere (and who can entertain a doubt on the subject?) we may point to our changes, our reforms, our improvements also.

Few things have struck me as being more remarkable than the simplicity of appliances and dressing in modern surgery among the best-class practitioners. This arises, I believe, from a better appreciation of the powers of nature, and a more humble idea of our own as to forcing that which can only come in time. It is, perhaps, in the increased knowledge and better treatment of wounds that the true philosophy of surgery has been most evinced in modern times. The days of the "secret dressings" and of "sympathetic powders" have passed away; and such a man as Colbatch, whom John Bell designated as a "respectable quack," or a pretender like Sir Kenelm Digby, were he even, like that famous man, secretary to a king, would have no influence on the profession, and little on the public, now-a-days. Yet Digby, had he belonged to our profession, would nearly have been a philosophic surgeon. If, after bringing the edges of a wound into accurate contact, and keeping them so by simple means, instead of affecting mystery and enacting the part of a mountebank, he had told his patient that he had done all that man could, and that nature and time would do the rest, he would have struck the key-note of that which constitutes, in my opinion, a great feature in modern practice. The secresy and sympathy consisted, in reality, in simplicity; and it remained for John Hunter and for what John Bell called "the London school" to give us our present views on such subjects.

Professor Hughes Bennett, of Edinburgh, has in recent years insisted much on what he calls "rational medicine," the term evidently implying the existence of a converse practice. It is not for me, in my present position, to say much about the practice of physic, but I do not hesitate to say that there is room for "rational surgery" to make useful way. "I cure"

or "we cure" is too much in our vocabulary; and it would be more in accordance with the knowledge we possess of nature's actions were we to affect less in this respect, whilst there is a broad margin on which the guiding head of the surgeon might take full credit. It has, indeed, been truly said that surgery is the handmaid to nature; and when the service is judiciously administered our work appears in the greatest perfection. Nature, in many of her inscrutable ways, does that which offends our common humanity; she brings us fevers, atrophies, consumptions, and cancers, over which we have but little control. Livingstone has told us that in parts of Africa where the lights of civilization have not yet appeared, most of those diseases which are at present the scourge of Europe have not yet been seen. May it not be that our boasted civilization has brought upon us many of those "evils" which, with a sort of negative consolation, we say, in poetic language, that "flesh is heir to?" Does not the very style of living interfere with nature's healthful actions in civilized man? Who in these islands can boast of success in lithotomy such as that obtained by our surgeons who practice in Asia? Were cases of elephantiasis scroti prevalent among us, is it likely that we could boast of saving 22 patients out of 24 operations? Yet such success has been recently recorded by Prof. Ballingall, of the Grant Medical College, Bombay. With all deference to our friends and contemporaries, it can not be admitted that this success comes from superior skill or dexterity; it is from the subject on which they work—the nearest approach to perfect nature, irrespective of what we fondly call civilized habits.

In speaking of wounds, I should not be doing justice to my own views and experience, nor to the efforts of others, were I to omit reference to the more common use of stitches than was sanctioned some thirty or forty years ago. When early and perfect union is desired in a line of considerable length, they far surpass other methods, and when judiciously applied (possibly in many instances with a due share of additional support) they are of the utmost value. Throughout my experience I can not say that I have seen the slightest evil arise from them, whilst the best possible good has often been derived. In fact, some of the greatest triumphs of modern surgery are associated with this simple mechanical process; for how else could so much have been done with those vesico-vaginal fistulæ which so baffled our forefathers, and are now so amenable to skillful operative management? How else could the operation for cleft palate have been successfully accomplished?

How else could we have dared to lay open the walls of the abdomen to the extent of six, twelve or fifteen inches? Much has been said in recent times of the superiority of the wire over thread as the material for the stitch; but for my own part I deem the subject of comparatively little importance, whilst I do not hesitate to proclaim my preference of common silk thread for general use.

Until within the present century there was no positive remedy for stone in the bladder but a painful and dangerous cutting operation. The highest talent, skill, and manipulative dexterity have been evoked to set aside the dangers of that proceeding. Surgeons have cut twenty, thirty, fifty patients, losing perhaps only one; but a more extended experience has had the effect of bringing the average of mortality down to the certain loss of one in six or ten. Men have vainly prided themselves on their success—some because of the peculiar shape of a knife; some on the supposition that they have operated more dexterously than others; and superior success has ever been claimed on account of a special prayer and appeal to the Almighty just before commencing! We know full well how in the mysterious ways of Providence, man's best efforts have failed; his holiest aspirations have seemingly been thwarted.

Happily we of the present day have lived to see the perils and uncertainties of lithotomy set aside in a large number of instances by the less formidable and possibly more successful proceeding of lithotrity. The development of this operation has been within our own time. It is of foreign origin, and British surgeons have taken slowly to it. Until within these twenty years it was practiced by few, but latterly it has come into more general use; and if patients would but apply at an early date, when the stone is small, the judicious employment of this operation would go far to supersede the use of the knife and make lithotomy exceptional. As evidence of the high and useful character of the operation, it has been applied alike to the peasant, the artizan, and the wearer of a crown. Whilst we do all honor to the labors of Gruithuisen, Le Roy, Heurteloup, Costello, and especially Civiale, in developing this proceeding, it is worthy of note that the essential features of the instrument now in use—namely, the male and female blades, with the sharp curve at the end where the crushing is to be effected, and the screw force for that purpose—are of English origin, having been devised by the late Mr. Weiss, our celebrated instrument maker.

For my own part, I am almost disposed to consider that the treatment of distortions by divisions of tendons, muscles, and fasciæ—a treatment founded on a better appreciation than formerly of anatomy, physiology and pathology—constitutes, perhaps, the most striking example of modern improvement which I could bring to your notice. I take great pleasure in referring to a case, treated by one of our provincial surgeons—Dr. Wiblin, Southampton—who in the discharge of his duties, like many others of his fellow-laborers, undertakes the treatment of most ailments that come within his cognizance with energy, skill, and success, such as may well be admired—possibly envied—by his metropolitan cotemporaries. Cutaneous puncture and subcutaneous division with a narrow blade, so as to prevent the access of air, make Stromeyer's name worthy of honor in all time to come; and the development of the new tendon in some of these cases is a fine illustration of what Nature will do where man judiciously interferes with some of her imperfect works.

How hopeless was our practice for strabismus in former times! We neither knew the cause, nor the means of cure. In the generality of such cases the division of the internal rectus of the eye restores the symmetry of these important and attractive organs. Here the simplicity of the idea almost leads us to overlook its magnitude and scientific character. The illustrious Roux thought his achievement great when he could close by operation the cleft palate as if it were a hare-lip, and be successful in securing union in two cases out of every three operated on. It is my intention to show you in some future lecture how, by division of the levator palati on each side, the operation may be rendered almost as certain in its results as that for fissure in the lip, and that the average of failures is about 1 in 27 or 30.

The skill with which raw surfaces are made and approximated says much for modern progress. Our plastic operations are more marvelous than ever entered the imagination of Taliacotius, or the poetic mind of Butler. The almost fabulous transplanting of one part of the body to a distant surface has been realized. The skin on the back of the neck has been lifted forward to supply a deficiency in front, and a portion of the skin of the abdomen has actually been made to do permanent duty on the forearm. Amongst plastic operations, and as illustrative of the value of union by the first intention, I may here refer especially to reparations on the face, and to the closing of wounds and unnatural openings in the urinary organs and parts of generation, particularly in the female. A word of praise in these departments is justly and specially due to our transatlantic brethren, and amongst ourselves there are many whose triumphs in these cases do the utmost credit to modern surgery.

The application of the stethoscope to surgical diagnosis, the exclusive use of the microscope in pathology, the invention of the laryngoscope and its recent application in practice, are all interesting features in modern surgery. The ophthalmoscope, too, is one of the most ingenious and clever inventions for which surgery is indebted; nor can there be a doubt that, in special cases, the speculum is also of vast service. But I must leave it to greater enthusiasts, and those more skilled than myself, to dilate upon the marvels divulged by these instruments and to fix upon their relative values as additions to the surgery of the present century.

Ophthalmic surgery has made wonderful strides within our own time; but I do not profess myself competent to dwell on such a theme. It is pleasing to see that those who excel in this department, particularly amongst ourselves, are gentlemen who, from their education and competency, are fitted to hold the highest places in general surgery, and that many of them have held, and now hold, the foremost rank in our profession. Let me here express a hope that some future professor in this chair may be able to say as much for all who may devote themselves to the specialties of modern custom.

Excisions, or resections—the words seem synonymous—have claimed a large share of modern attention; for although we owe to the last century many such proposals and several examples, it is within the present that much has been said about them. But time presses, and I shall conclude my present address by reference to some matters which need not be dwelt upon in other lectures. Of these, that of anæsthesia may be deemed the most remarkable. No single appliance in surgery can, in my opinion, be compared with it; for, although before its discovery most, if not all, of the great achievements of our art had already been accomplished, the amount of suffering which can now be set aside enables us to relieve surgery of much of its horrors, and to exclude from the patient's senses that which was anguish, suffering and torture; whilst, generally, it permits the surgeon to perform his duty with a serenity of thought and action quite unknown to his predecessors. On this subject America again must have the palm of precedence. There sulphuric ether still holds the first place as the anæsthetic agent; whilst with us chloroform, whose influence was first observed and made public by one of our contemporaries, is considered the most useful. Not long ago Dr. Marion Sims, with laudable enthusiasm, claimed for metallic stitches the honor of being, in our profession, the greatest discovery of the nineteenth century. Few surgeons of practical experience, however, will endorse this. I see nothing which has transpired in the present century which, in magnitude or importance, can compare in our annals with anæsthesia; and in my mind, it ranks in value to mankind scarcely less than the results of the labors of Harvey and of Jenner.

We congratulate ourselves that we have been permitted to live in times when man has displayed his mastery over steam and electricity, and with us and our special profession there have been agencies at work whose usefulness may be said to be literally beyond calculation. I allude to the improved facilities for education, to our social professional customs, to the medical press, and our own special literature. Our schools have increased in number; our great public hospitals associate more extensively than ever, education with charity; our handbooks, our works of reference, our means for learning, our appliances for teaching are beyond compare; and facilities for studying anatomy have, by a wise legislation, been placed lawfully within reach. Our societies and professional gatherings have encouraged and facilitated the diffusion of knowledge; man meets man face to face, thoughts flash almost simultaneously from brain to brain; and there is no longer a difficulty with those in places distant from a metropolis to find out even some roundabout way of communicating interesting or useful knowledge to the profession. A surgeon to a Liverpool hospital in the present day need not, as Park did in 1782, address himself to a leading hospital surgeon in London to give currency to his aspirations; nor need the Moreaus of our day keep their originality under the "cold shade" of an academy or a corporation. Besides the facilities for individual and independent publication, there are our quarterly, monthly, and weekly journals to carry knowledge to the ends of the earth. We pride ourselves in this country on the liberty of the press; we fondly call it our fourth estate; politically and professionally it may be called the pulse of the public mind; and amongst ourselves in our own time it beats with a healthy vigor, indicative of all those changes for the better which I have endeavored to sketch, although I fear but feebly, within the limits of a single lecture.—*London Lancet.*

